# Louisville Law School

**Published:** 1846-09

**Authors:** 


### LOUISVILLE LAW SCHOOL.

The trustees of the Louisville University have wisely resolved to organize a Law Department, the professorships in which have been created and filled. The following gentlemen have been elected, to wit:

Hon. Henry Pirtle, LL.D., Professor of Constitutional Law, Equity and Equity Pleadings, and Commercial Law.

Garnet Duncan, Esq., Professor of the Science of Law, including the Common Law and its History, and the Laws of Nations.

Preston S. Loughborough, Esq., Professor of the Law of Real

Property, the Practice of Law, including Actions, Pleadings, and Evidence, and the Criminal Law.

These gentlemen are well known in all parts of our country as among the most able and learned jurists in our Commowealth, famed for the ability of its bar; and we congratulate our young countrymen of the law that the services of such teachers are henceforth to be at their command. Aside from their experience and legal erudition, the examples of dignity, probity, and purity afforded by the lives of these eminent men will be of incalculable value to their pupils. We predict for the Law department of the University of Louisville a rapid rise and prosperous career. The newly elected professors have made arrangements for opening the department in November, and a full course of lectures will be delivered during the approaching winter.

---

LOUISVILLE SUMMER SCHOOL.

